# The Etiology, Prevention and Blood Serum Diagnosis of Cancer of the Stomach and Intestines

**Published:** 1905-02

**Authors:** 


### THE ETIOLOGY, PREVENTION AND BLOOD-SERUM DIAGNOSIS OF CANCER OF THE STOMACH AND INTESTINES.*

Dr. Kelling, who, after thorough experimental work, has come to the conclusion that malignant growths represent nothing else than the product of development of foreign, especially embryonic animal cells within the human body, has continued his untiring research with apparently satisfactory results.

In experimenting on twenty-one cases of malignant growths in man and one carcinoma mammæ in a dog, he proved by the biochemical method the presence of chicken-albumen in nine cancerous growths of men, and he takes it for granted that these nine growths, which were all situated in the alimentary tract, were caused by the parasitic development of embryonic chicken cells.

In four other cases, viz., a mamma-carcinoma of a woman, a mamma-carcinoma of a dog, an ovarian sarcoma of an old lady, and a teratoma of the testicle in a young man, he ascertained the presence of pig-albumen and concluded that these tumors were caused by the development of embrionic pig-cells.

*By Dr. Georg. Kelling, (Weiner Med. Wochenschrift, No. 37-38, 1904. Ref. Muenchener med. Wochenschrift, No. 43, 1904.)

He further strengthens his theory by the assertion that he was able by the same biochemical method to make the diagnosis of malignant growth in the living subject even in cases, where there were no objective signs of this disease, another diagnosis having been made.

If the malignant growth really consists of animal cells, like chicken cells, for instance, the afflicted body, as long as there is no cachexia or pronounced lack of nutrition, produces the so-called precipitin-albumen. This product precipitates this kind of albumen.

From this we deduct that if we can prove the presence of an animal precipitin-albumen in the serum of a man's blood, a malignant growth, consisting of cells of the same animal, must be present somewhere in that human body. Out of fifteen patients suffering from malignant growths in different portions of the alimentary tract, he found present in the blood-serum of eight, precipitin chicken-albumen, and precipitin pig-albumen in two. In other words, Dr. Kelling considers the presence of an animal precipitin albumen in the human blood-serum pathognomonic for malignant growths. In a man thirty-four years old, where the diagnosis of chronic catarrh of the stomach was made and no sign of malignant growth could be made out by the usual method, Dr. Kelling was confident enough to advise laparotomy, because he had found precipitin chicken-albumen in the patient's blood-serum, and on operating carcinoma of the smaller curvature of the stomach and the omentum minus was discovered.

The author draws the conclusions that his method of examining the blood-serum for animal precipitin albumen is of great value in the diagnosis of malignant growth, that the prophylaxis of malignant growth is much easier than the prevention of infective diseases. It consists in avoiding raw eggs and meat and in the removal of dogs and cats as a frequent source of infection from our home. All the wombs of slaughtered animals should be removed and confiscated by the slaughter-house inspection, and not even be used for feeding dogs and other animals.

